# The Book World of Medicine and Science

**Published:** 1899-04-29

**Authors:** 


### The Book World of Medicine and Science.

Saline Injection in Abdominal Operations. By G. A. Hawkins-Ambler, F.R.C.S. (Edinburgh: Neill & Co., 1899. Pp. 21.)

This interesting pamphlet is a reprint of a paper written by Mr. Hawkins-Ambler for the *Liverpool Medico-Chirurgical Journal*. Its scope is sufficiently indicated by the title, and its tone is eminently practical. But some of the author's contentions are deserving of special attention. Mr. Ambler desires, and so we doubt not does every practical surgeon, to make the use of hot irrigation an intelligent procedure rather than a part of an unreasoning routine. He denies, however, the value of irrigation as a means of cleansing the peritoneum, and is inclined to regard it as a means often of diffusing sepsis. He doubts the hæmostatic power of water warmed within such thermometric limits as may for the peritoneum be regarded safe. Mr. Hawkins-Ambler believes, however, that the irrigation of the peritoneum with warm saline solution is, in virtue of the peritoneum's powers of absorption, perhaps the readiest way of averting shock and of preventing symptoms, such as thirst, which are consequent on drain of the body fluids. He also believes—and with apparent good reason—that the free use of irrigation during and after abdominal section tends to prevent the formation of adhesions. In conclusion, we may say that Mr. Hawkins-Ambler's remarks are interesting and cogent, and that his opinions deserve the careful attention of those best qualified to criticise them.

The Chemical and Biological Analysis of Water. By T. H. Pearmain and C. G. Moor, M.A.Cantab. (London: Baillière, Tindall, and Cox. 1899. Pp. 172. Price 5s. net.)

We had occasion some few months ago to criticise—and not altogether favourably—a work on "General Bacteriology" by the authors of the manual now before us. But of this book we are glad to speak in terms of less qualified praise. It is the second part of a series dealing with the analysis of food and drugs, and from its nature affords Messrs. Pearmain and Moor ample opportunity of drawing on their stores of special experience. Without having written an exhaustive treatise, they have given us in these 172 pages an adequate account of the methods of water analysis; and, on the whole, have held the balance fairly as between the chemist and the bacteriologist. A sufficient account is also given of the larger organisms that may be found in water, but the illustrative drawings are not of great merit. Naturally the bacteriological section deals fully with the differentiation of B. coli communis from B. typhosus. But the organism of cholera is somewhat unworthily treated of. Missing as we do any account or analyses of natural mineral waters we are a little surprised to find such elaborate analyses of various commercial products—as far as we can judge, somewhat arbitrarily selected. Nor can we think it wise, in a book of this nature, to pass encomiums on articles in the market in such set phrases as lend themselves to ready quotation.

An Experimental Research into Surgical Shock. By G. W. Crile, A.M., M.D. (Philadelphia: J. B. Lippincott. 1899. Page 160. Price 12s. 6d.)

Dr. Crile—an American surgeon—proposed to himself some time ago, as we gather, to elucidate some of the physiological details of surgical shock. To this end he conducted experiments—in London and in America—on 148 dogs. The records of the experiments and the conclusions drawn are here presented to us. We have little sympathy with those who wish to hamper experimental research, but, as a matter of scientific criticism, are bound to comment somewhat unfavourably on Dr. Crile's work. In the first place, every medical student is aware how profoundly the phenomena of shock are modified by anæsthesia and how profoundly anæsthetics modify the normal phenomena of blood pressure and respiration. Since then, as Dr. Crile's energies were practically restricted to the observation of the variations in blood pressure and respiration produced in dogs—as he assures us, all anæsthetised—by various mutilations, we are greatly surprised that in the notes practically no information is afforded us of the anæsthetic employed in each case, of the quantity used, or of the duration of its employment. In several experiments we are told curare and morphia were employed. We are not told the dose administered, and whether or no any other anæsthetic agents were used. In every case a preliminary tracheotomy was performed; in many cases artificial respiration was necessary. The deductions made therefore from the elaborate manometric tracings must be accepted with more reservation than Dr. Crile deems necessary. Again, we are totally unable to conceive what bearing on traumatic shock, as we know it clinically, have the results of pouring boiling water into dogs' abdominal cavities or even the forcible dilatation of the vaginas of bitches. Certainly, after "smashing the jaws," "extirpating an eyeball and roughly manipulating the socket," after crushing thoraces, and "roughly handling" peritoneum and bowels for half an hour, Dr. Crile is is a position to assure us that clean cuts with a knife produce less shock than rough manipulations and lacerations. Indeed, Dr. Crile seems a little fearful that his research has been unproductive, for he states that the prevention of shock is best accomplished "by taking into account all the known physiologic (*sic*) functions . . . of the body in a way that would suggest itself to any practical surgeon." The final utterance of this physiologist is cryptic, though possibly less profound than it appears: "The result of action is reaction; of rest is restoration." We repeat, we have criticised this book purely from a scientific standpoint. It contains evidence of much patient work, of much enthusiasm, of much ingenuity. It is a pity that such qualities should have been diverted from clinical study to such an unprofitable research as this on surgical shock.

The City of London Directory for 1899. With a Coloured Map on a Large Scale. (London: W. H. and L. Collingridge. 1899.)

This well-known publication retains all the old features by aid of which it has attained its unique position as a guide to all that pertains to the City of London as well as a mere directory. It is to be noted that as the result of the great destruction of property at the time of the Cripplegate fire a considerable number of streets have had to be entirely reconstructed, and that considerable changes have taken place in the occupiers of the various buildings. These and other alterations have had to be recorded, as well as several changes in the names of streets. The biographical section is fully illustrated with portraits of City dignitaries, and it is impossible to speak too highly of the splendid map which accompanies the volume.

